# Altered Gene Transcription in Human Cells Treated with Ludox^®^ Silica Nanoparticles

**DOI:** 10.3390/ijerph110908867

**Published:** 2014-08-28

**Authors:** Caterina Fede, Caterina Millino, Beniamina Pacchioni, Barbara Celegato, Chiara Compagnin, Paolo Martini, Francesco Selvestrel, Fabrizio Mancin, Lucia Celotti, Gerolamo Lanfranchi, Maddalena Mognato, Stefano Cagnin

**Affiliations:** 1 Department of Biology, University of Padova, Via G. Colombo 3, Padova 35121, Italy; E-Mails: caterina.fede@unipd.it (C.F.); chiara.compagnin@unipd.it (C.C.); paolo.martini@unipd.it (P.M.); lucia.celotti@unipd.it (L.C.); gerolamo.lanfranchi@unipd.it (G.L.); 2 Centro di Ricerca Interdipartimentale per le Biotecnologie Innovative—CRIBI Biotechnology Centre, University of Padova, Via U. Bassi 58/B, Padova 35121, Italy; E-Mails: caterina@cribi.unipd.it (C.M.); benny@cribi.unipd.it (B.P.); bcelegato@gmail.com (B.C.); 3 Department of Chemical Sciences, University of Padova, via Marzolo 1, 35131 Padova, Italy; E-Mails: francesco.selvestrel@gmail.com (F.S.); fabrizio.mancin@unipd.it (F.M.)

**Keywords:** nanoparticles (NPs), cell toxicity, microarray gene expression, pathway analysis

## Abstract

Silica (SiO_2_) nanoparticles (NPs) have found extensive applications in industrial manufacturing, biomedical and biotechnological fields. Therefore, the increasing exposure to such ultrafine particles requires studies to characterize their potential cytotoxic effects in order to provide exhaustive information to assess the impact of nanomaterials on human health. The understanding of the biological processes involved in the development and maintenance of a variety of pathologies is improved by genome-wide approaches, and in this context, gene set analysis has emerged as a fundamental tool for the interpretation of the results. In this work we show how the use of a combination of gene-by-gene and gene set analyses can enhance the interpretation of results of *in vitro* treatment of A549 cells with Ludox^®^ colloidal amorphous silica nanoparticles. By gene-by-gene and gene set analyses, we evidenced a specific cell response in relation to NPs size and elapsed time after treatment, with the smaller NPs (SM30) having higher impact on inflammatory and apoptosis processes than the bigger ones. Apoptotic process appeared to be activated by the up-regulation of the initiator genes *TNFa* and *IL1b* and by *ATM*. Moreover, our analyses evidenced that cell treatment with Ludox^®^ silica nanoparticles activated the matrix metalloproteinase genes *MMP1*, *MMP10* and *MMP9*. The information derived from this study can be informative about the cytotoxicity of Ludox^®^ and other similar colloidal amorphous silica NPs prepared by solution processes.

## 1. Introduction

Nanotechnology allows the manipulation and organization of elements in a reduced dimension scale (nanometer scale range) permitting the development of nanomaterials with enormous potential impact on different applications in biotechnology, medicine, diagnostic, food and material science. The great promise of nanotechnology is to improve the quality of products, however, there are many questions about the health risks that may be associated with exposure to nanomaterials. Moreover, it is not clear if the materials usually safe can become dangerous when reduced to the nanoscale because it is assumed that the laws of chemistry and physics work differently when the particles are infinitely small [1]. Nanomaterials can be generated from parent material (transitional metals, silicon, carbon, and metal oxides) in different shapes: spheres, rods, wires, and tubes [2]. Some known NPs tend to agglomerate and fall to the ground, while, the latest nanomaterials are often coated to prevent the agglomeration causing a longer volatility and, therefore, becoming more easily inhalable. In this context, silica NPs have found extensive applications in industrial manufacturing and chemical industry, as additive used as a flow agent in powdered foods or to adsorb water in hygroscopic applications, as fining agents for wine, beer, juice, as additives to cosmetics, printer toners, drugs and pharmaceutical products [3]. Considering the increasing exposure to silica NPs, studies are required to assess their impact on human health. Because of their very small size, NPs can enter cells interacting with intracellular structures and molecules [4] and causing health problems [5,6,7,8,9]. Different mechanisms are the basis of nanomaterials’ toxicity, but the most pronounced arise from the production of excess reactive oxygen species (ROS) that contribute to the generation of oxidative stress, mitochondrial perturbation, inflammation, and endothelial dysfunction [10,11,12]. In general, the physicochemical features of engineered NPs influence their toxicity [12]: the smaller the dimensions easier is the ability to cross tissue and cell membranes allowing the interaction with cellular components and working as molecular switches [13]. Ludox^®^ NPs have chemically active surfaces that bond to other silica particles or oxygen-containing surfaces. Most applications of Ludox^®^ colloidal silica depend on the high surface area and reactivity of the suspended particles, and on the chemical inertness, excellent refractoriness and low coefficient of expansion of these particles when dry. Some of the Ludox^®^ NPs applications are as coating agents for plastic films or for photographic and duplicating paper, antisoil, adhesion or wetting promotors, antislip, reinforcing agent for latex, silicon wafer polishing, soil retardants and rug shampoos, package fractionizing and high temperature binders. Colloidal silica may cause a tissue response in the lung (pneumoconiosis) if mists or dusty dried particles are inhaled. The lung cell culture models should be used in nanotoxicity studies to investigate the most affected system by volatile NPs and for this purpose, human alveolar epithelial cells (A549 cell line) have been frequently used [14]. Common *in vitro* methods (cytotoxicity or cell viability assays, apoptosis or necrosis detection) allow the production of specific and quantitative measurements of nanotoxicity, but provide little information about the mechanisms or causes of cellular toxicity and death.

Omics science applied to nanotechnology is now emerging as an attractive tool to address the still unanswered questions dealing with nanoparticle-induced toxicity in living systems. The unique advantage provided by “omic” techniques (such as, two dimension DIfference Gel Electrophoresis: 2D-DIGE, Liquid Chromatography Mass Spectrometry: LC-MS, microarrays) is to get information on the systems level considering molecular interactions and pathway alterations induced by and related to NPs. Omics approaches should allow the identification of biomarkers to monitor the effects of NP exposure. In comparison to other health related problems (e.g., tumors, skeletal muscle pathologies), genome wide approaches were little used to understand mechanisms underlying the nanotoxicological effects. Protein expression profiles allowed the identification of an early acute response, not associated with general physiological damage, due to treatment of rats with SiO_2_ [15], while MAPK pathway and cell cycle alterations were evidenced in A549 cells treated with CuO NPs [15]. All genome wide analyses performed to detect effects of NPs in treated cells [16,17,18,19,20,21,22,23,24,25,26] are based on the identification of differentially expressed genes that represent the starting point of a highly challenging process of result interpretation in which a gene-by-gene approach is often used. The lists obtained are highly dependent on the statistical tests adopted and on the threshold used to declare a gene significant. This variability has raised substantial criticism concerning the reproducibility of array experiments. Several studies have demonstrated greater consistency in array results using gene set approaches, rather than single gene approaches [27,28,29], indicating that there is greater reproducibility of the main biological themes than of their single elements. A gene set is defined as a set of genes that are functionally related. Gene sets are usually identified based on *a priori* biological knowledge (see, for example, Gene Ontology “GO” [30], the Kyoto Encyclopedia of Genes and Genomes “KEGG” [31] and Reactome [32]). In this work, we used the microarray gene expression profiling to identify gene sets altered in human lung cancer cells (A549) in relation to SiO_2_ NPs of two different sizes (SM30 and AS30) and to the recovery time after exposure. By integrating gene sets and gene-by-gene approaches we evidenced the activation of matrix metalloproteinases genes *MMP1*, *MMP10* and *MMP9* and immune and apoptosis processes in response to smaller Ludox^®^ silica nanoparticles (SM30).

## 2. Experimental Section

### 2.1. Nanoparticle Characterization

Ludox^®^ silica NPs of two different sizes, AS30 and SM30, were obtained from a commercial source as 30 wt % suspensions in H_2_O. The nanoparticle suspensions were diluted with ultrapure (Milli-Q Merck Millipore, Billerica, MA, USA) water to the desired concentration (30–40 mg/mL), extensively dialyzed into a 75 mL Amicon ultrafiltration cell, equipped with a 10 kDa regenerated cellulose membrane, and finally filtered with 0.22 μm Durapore membrane. NP concentration in the purified sample was determined by weighing a dried aliquot of the solution. Transmission electron microscopy (TEM) images of the particles were obtained with a FeiTecnai 12 transmission electron microscope (FEI, Hillsboro, OR, USA) as previously described [33]. Dynamic light scattering (DLS) measurements were performed with a Zetasizer NanoS (Malvern, Malvern, Worcestershire, UK) equipped with a thermostatic cell holder and an Ar laser operating at 633 nm.

### 2.2. Cell Line and Treatments

The human cell line A549 (lung adenocarcinoma) was obtained from the American Type Culture Collection (American Type Culture Collection, Rockville, MD, USA) and maintained in F12-K medium supplemented with 10% heat-inactivated Fetal Calf Serum (FCS), 38 units/mL streptomycin, and 100 units/mL penicillin G under standard culture conditions and during the post-treatment recovery. Cells were kept at 37 °C in a humidified atmosphere containing 5% CO_2_.

To evaluate the cytotoxicity induced by Ludox^®^ NPs, the cells were plated and allowed to attach for 24 h. Then, NPs were diluted to appropriate concentrations and immediately applied to the cells. We used a short incubation for 2 h in serum-free medium, followed by a post-treatment recovery of 3 or 22 h in complete medium (10% FCS). We selected these treatment modalities because DLS measurements showed that NPs aggregate in the presence of serum, and preliminary cell viability tests suggested that 2 h is the maximum time interval of culture in medium without serum tolerated by cells. For long incubation times, we supplemented culture medium with 3% serum, which represents the lowest percentage suitable for maintaining the cells for to 72 h without suffering, in accordance with our previous observations [34]. For cytotoxicity tests, the dialyzed NP stock suspensions were diluted with ultrapure water (5 mg/mL); the pH was adjusted between 7.3 and 7.5 with 1 M HCl, and the suspensions were sterilized by 0.22 μm filtration (control experiments confirm that such operations do not alter the nanoparticles’ concentration). The diluted solutions were prepared immediately before use. NPs concentrations (0.005–0.6 mg/mL) were chosen to evaluate the dose/survival according to the treatment conditions. Control cells underwent the same steps of treated cells except for NP exposure.

### 2.3. Assessment of Cytotoxicity and Apoptosis Detection

Analyses were performed as described in [33]. Briefly, 8 × 10^3^ cells/cm^2^ were seeded in triplicate in 96-well plates (200 μL/well). After 24 h, the culture medium was removed, and the cells were incubated for 2 h with 150 μL of medium, without serum, containing different concentrations of AS30 or SM30 NPs. After recovery time (3 or 22 h in complete medium with 10% of serum), the cells were incubated for 60–90 min in the dark with 20 μL of the MTS reagent diluted in 100 μL of serum-free medium. The absorbance of formazan product was recorded at 490 nm with a microplate reader (Spectramax 190, Molecular Devices^®^, Sunnyvale, CA, USA). A clonogenic assay was performed by seeding 2–4 × 10^4^ cell/cm^2^ in 6-cm culture dishes allowing their attachment overnight. After the treatment cells were harvested by trypsinization, and counted by trypan blue dye exclusion. Next 10.2 cell/cm^2^ were plated in culture dishes, and after 7–14 days at 37 °C, the colonies were stained with 0.4% crystal violet and counted. Only colonies containing more than 50 cells were scored as survivors. Cell survival was calculated as a percentage of cloning efficiency (CE) of treated cells over CE of control cells. Induction of apoptosis in treated cells was performed by the Annexin-V-FLUOS Staining Kit (Roche Applied Science, Basel, Switzerland). After the treatment, cells were detached and centrifuged at 200× *g* for 5 min. The pellet was resuspended in 100 μL of Annexin-V-Fluos labeling solution and incubated for 10 min at 37 °C. Samples were analyzed by flow cytometry with a FACSCantoTM II flow cytometer (BD Bioscences, San Jose, CA, USA).

### 2.4. Assessment of Microarray Experiments

RNA extraction was performed using TRIzol (Invitrogen, Carlsbad, CA, USA) according to the manufacturer’s protocol. All samples were quantitated using a NanoDrop ND-1000 spectrophotometer; RNA quality was then analyzed using the Agilent Bioanalyser 2100 (Agilent, Santa Clara, CA, USA) (Agilent RNA 6000 nano kit; RIN at least 7 accepted). 1 µg of total RNA was labeled with “Agilent One-Color Microarray-Based Gene Expression protocol” according to the manufacturer’s protocol. The synthesized cDNA was transcribed into aRNA and labeled with Cy3-dCTP. Labeled aRNA was purified with RNeasy Mini columns (Qiagen, Valencia, CA, USA). The quality of each aRNA sample was verified by total yield and specificity calculated with NanoDrop ND-1000 spectrophotometer measurements (Nanodrop, Wilmington, DE, USA). Labeled aRNA (1.65 μg) was used in each reaction and hybridization was carried out at 65 °C for 17 h in a hybridization oven rotator (Agilent). The arrays were washed using Agilent Gene expression washing buffers and Stabilization and Drying Solution as suggested by the supplier. Slides were scanned on an Agilent microarray scanner (model G2565CA) and Agilent Feature Extraction software version 10.5.1.1 was used for image analysis. Gene expression data were performed on three biological replicates for each condition and are available in the GEO database with the accession number: GSE53700.

### 2.5. Microarray Data Analysis

Inter-array normalization of expression levels was performed with the quantile method [35] to correct possible experimental distortions. Normalization function was applied to the expression data of all the experiments, and the values for within-arrays replicate spots were then averaged. Feature Extraction Software, which provided spot quality measures, was used to evaluate the quality and reliability of the hybridization. In particular, the flag “positive and significative” (set to 1 if the spot had an intensity value significantly different from the local background and to 0 when otherwise) was used to filter out unreliable probes: the flag equal to 0 was to be noted as “not available (NA)” the spot intensity. Probes with a high proportion of NA values were removed from the dataset in order to carry out a more solid and unbiased statistical analyses. Thirty-three percent of NA was used as the threshold in the filtering process, and a total of 32,096 transcripts of 41,093 were used in the subsequent analysis. Gene expression data derived from cells treated with NPs were divided by the expression of the same gene in the corresponding control sample and then log transformed before the identification of differentially expressed genes. Differentially expressed genes identification (Significance Analysis of Microarray [36]; multiclass analysis with 5% FDR accepted), and PCA analysis were performed using the TMev suite [37]. Classes considered for the identification of differentially expressed genes were (a) cells treated with SM30 for 3 h of recovery time, (b) cells treated with AS30 for 3 h and (c) 22 h of recovery time. Gene Ontology analysis of differentially expressed genes was performed according to DAVID web tool [38]. Cytoscape tool [39] in association with the Agilent Literature Search plug-in [40] was used to produce network of differentially expressed genes. Agilent Literature search software is a meta-search tool for automatically querying multiple text-based search engines (both public and proprietary: PubMed, OMIM: Online Mendelian Inheritance in Man, USPTO: United States Patent and Trademark Office) in order to aid biologists faced with the task of manually searching and extracting associations among genes/proteins of interest. In particular, searched genes can be contextualized and related to association terms of interest. We used following terms to associate gene symbols of up-regulated genes in SM30 treated cells (Supplemental Table S1) with specific context: “nanoparticles”, “nanotoxicology”, “cell toxicity”, “nanostructure”, and “nanomaterials”. *Homo sapiens* concept lexicon was used to resolve gene aliases and search engine “hits” was limited to ten per search engine per query line. The Agilent software converts into interactions searched terms linked by a verb. Resulted network (643 nodes and 2193 edges; Supplemental Figure S1) was analyzed using Network Analyzer [41] plug-in. Topological analysis by Network Analyzer was performed on undirected network (containing only undirected edges). Parameters considered were: (a) connected components; (b) number of neighbors; (c) network radius; (d) network diameter; (e) network centralization; (f) network density; (g) network heterogeneity and (h) clustering coefficient. Next we provide a brief description of the parameters that are described in [41,42] . All nodes of a network that are pairwise connected form a connected component. The number of connected components indicates the connectivity of a network—a lower number of connected components suggests a stronger connectivity. The network diameter is the largest distance between two nodes while the neighborhood genes of a node represent the number of nodes associated with it that can be normalized in the network density parameter. Network density value is comprised between 0 and 1. A network which contains no edges and solely isolated nodes has a density of 0. Parameters related to neighborhood are the network centralization [42] and the network heterogeneity [42]. The network heterogeneity reflects the tendency of a network to contain hub nodes while regarding network centralization if it has a value close to 1 the network resembles a star, whereas decentralized networks are characterized by having a centralization close to 0. The network radius is the minimum among the non-zero eccentricities (eccentricity is the maximum non-infinite length of a shortest path between a node and another node in the network) of the nodes in the network. The clustering coefficient is a ratio between the number of edges between the neighbors of a node and the maximum number of edges that could possibly exist between the neighbor nodes in the network. The clustering coefficient of a node is always a number between 0 and 1. The network clustering coefficient is the average of the clustering coefficients for all nodes in the network.

Supervised pathway analysis was performed by Gene Set Enrichment Analysis (GSEA) [43] and CLIPPER Analysis [44] implemented in the Graphite web tool (accessed on March 2014) [45]. This is a public web server for the analysis and visualization of biological pathways using high-throughput gene expression data. The aim of this type of analyses is to identify groups of genes with coordinated expression changes differentiating biological conditions. GSEA analysis allows determining whether an a priori defined set of genes shows statistically significant, concordant differences between two biological states. In this analysis we set at 10 the minimum number of genes in common between experimental data and pathways to compare, considering the expression of cells treated with SM30 and AS30 NPs with the smaller recovery time (3 h). CLIPPER algorithm accounts in the pathway analysis topological structure of the pathway to select it on the base of means significantly different between experimental conditions. Also in this case we set at 10 the minimum number of genes that have to be mapped on the analyzed pathways.

### 2.6. qRT-PCR Experiments

Total RNA (1 µg) was retrotranscribed with ImProm-II Reverse Transcription System (Promega, Madison, WI, USA). qRT-PCR was performed with the GoTaq qPCR Master Mix (Promega) and gene-specific primers for *MMP1*, *MMP10*, *TNFa*, *IL1b*, *ATM* genes and for *GADPH* as reference (Table 1). qRT-PCR reactions were always performed in triplicates according following PCR cycle: 95 °C for 2 min; 95 °C for 15 s and 60 °C for 1 min for 40 cycles; 72 °C for 1 min. The relative expression levels were calculated using the comparative delta CT (threshold cycle number) method (2^−ΔΔCT^) implemented in the 7500 Real Time PCR System software (LifeTechnologies, Carlsbad, CA, USA).

**Table 1:** Primers for qRT-PCR.

Primer Name	Sequence
MMP1 forward	AGAGAGCAGCTTCAGTGACA
MMP1 reverse	CTTGAGCTGCTTTTCCTCCG
MMP10 forward	TTGACCCCAATGCCAGGAT
MMP10 reverse	CCCCTATCTCGCCTAGCAAT
TNFa forward	AGTGCTGGCAACCACTAAGAA
TNFa reverse	AGATGTCAGGGATCAAAGCTG
IL1b forward	TACTCACTTAAAGCCCGCCT
IL1b reverse	ATGTGGGAGCGAATGACAGA
ATM forward	ACTGGCCAGAACTTTCAAGAAC
ATM reverse	TGCCCAGAATACTTGTGCTTC
GAPDH forward	TCCTCTGACTTCAACAGCGA
GAPDH reverse	GGGTCTTACTCCTTGGAGGC

## 3. Results and Discussion

### 3.1. Characterization of Ludox^®^ AS30 and SM30 Nanoparticles

**Figure 1:**
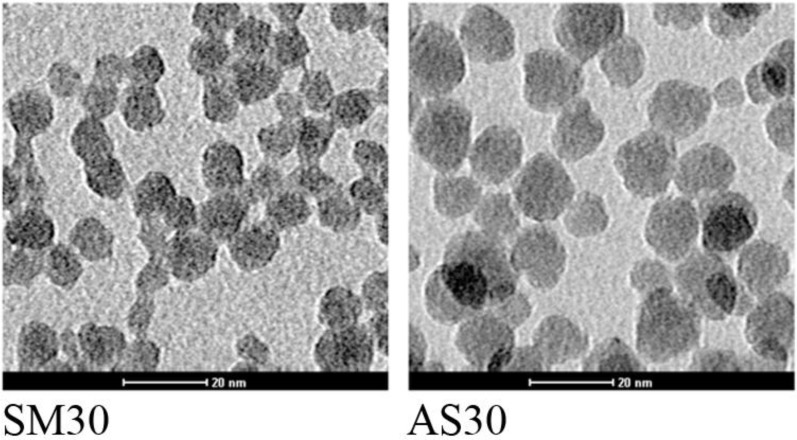
TEM images of SM30 and AS30 Ludox^®^ NPs. According to TEM analysis SM30 NPs have a diameter of 9 ± 3 nm while AS30 NPs have a diameter of 18 ± 3 nm. More information is provided in Table 2.

AS30 and SM30 Ludox^®^ NPs were selected for the present study because of their wide commercial applications. Dynamic light scattering (DLS) and Transmission Electron Microscopy (TEM) analyses were performed before and after the dialysis used to remove any possible contaminant confirming that the purification procedure does not alter the size and morphology of the NPs. The hydrodynamic diameters, obtained by DLS, were 20 ± 4 and 14 ± 4 nm for Ludox^®^ AS30 and SM30, respectively. The mean NP sizes determined by TEM micrographs were 18 ± 3 (AS30) and 9 ± 3 nm (SM30). DLS data were larger than the TEM radius: this is directly related to the solvation shell of molecules surrounding the NPs. Zeta potential of both NPs was negatively charged, −25.9 mV and −26.3 for Ludox^®^ AS30 and SM30, respectively, indicating that the two preparations of Ludox^®^ NPs have a similar stability. The data relative to Ludox^®^ NPs characterization are available in [33] (Figure 1, Table 2).

The behavior of NPs in pure water, culture medium, and in culture medium supplemented with low concentration (3%) of serum was investigated in [33]. We did not detect any aggregation either in PBS or in culture medium. On the other hand, Ludox^®^ NPs strongly aggregated when the medium was supplemented with small amount of serum (3%) and with NP concentrations ≤0.01 mg/mL. Such a behavior is consistent with the well-known protein flocculation ability of silica nanoparticles that is exploited in many applications as beverage clarification.

**Table 2:** NP properties.

NP Type	Counterion *	ζ Potential in PBS	DLS Diameter in PBS	Diameter from TEM in PBS	Surface Area *	pH *
**SM30**	sodium	−26.3 mV	14 ± 4 nm	9 ± 3 nm	345 m^2^/g	10.0
**AS30**	ammonium	−25.9 mV	20 ± 4 nm	18 ± 3 nm	230 m^2^/g	9.1

Note: With * are indicated data provided by Sigma-Aldrich, St. Louis, MO, USA.

### 3.2. Cytoxicity Induced by Ludox^®^ AS30 and SM30 Nanoparticles

**Figure 2:**
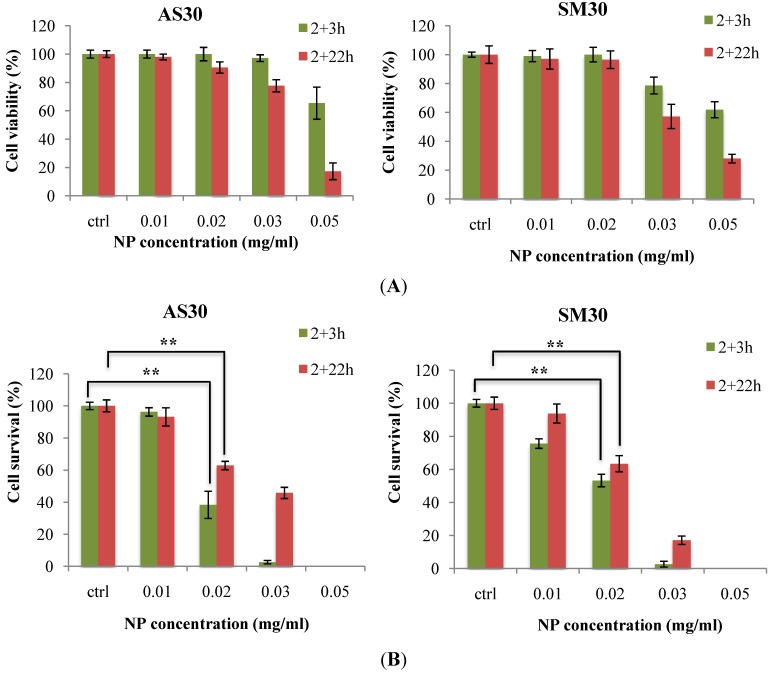
Cytotoxicity of Ludox^®^ NPs AS30 and SM30 in A549 cells was assessed in relation to NP concentration and recovery time after the treatment (3 or 22 h) by MTS assay (**A**, cell viability) and clonogenic assay (**B**, cell survival: cloning efficiency of treated/untreated control cells).

Notes: Data represent mean ± S.D. (3 ≤ *n* ≥ 12). The symbol of significance of cell viability/survival decrease is reported only for the dose 0.02 mg/mL used for gene transcription analyses (** *p* ≤ 0.01, *t*-test). All treatments with higher NP concentrations significantly increased cytotoxicity (Supplementary Figure S2).

Cytotoxicity induced by Ludox^®^ NPs was evaluated by the MTS assay, which measures the reduction of tetrazolium salts to formazan by metabolically active cells, and by the clonogenic assay based on number of colonies formed by single cells. The results showed that cell viability did not decrease after treatment with 0.02 mg/mL of the two NP types, neither after 3 nor after 22 h of recovery time (Figure 2A).

At higher doses (0.03, 0.05 mg/mL) cell viability was similarly altered by the treatment with both NPs. In contrast to MTS assay, the colony forming ability of A549 cells was significantly affected by the 0.02 mg/mL dose of Ludox^®^ AS30 and SM30 NPs at both recovery times after treatments (Figure 2B). Moreover, clonogenic assay showed that NPs decreased cell survival at very low concentrations; in particular, the treatment with 0.01 mg/mL after 3h from the treatment (Figure 2B). These results reflect the different sensitivity of MTS and clonogenic assays. The first is based on enzymatic activities detected either in viable and in senescent/dying cells, and the second on the retention, by only viable and healthy cells, of proliferation ability. The survival of A549 cells was similar at both time points after treatments, suggesting that this cell line did not recover proliferation ability during the post-treatment incubation. Results obtained by these assays showed that SM30 and AS30 NPs caused very similar levels of cytotoxicity in both treatment conditions, with the smaller SM30 NPs being slightly more effective.

### 3.3. Microarray Analysis: Differentially Expressed Genes

We analyzed the effects of Ludox^®^ SM30 and AS30 nanoparticles on gene transcription of A549 cells using microarray analysis. Gene expression profiles were performed on cells treated with NPs concentrated at dose of 0.02 mg/mL, which appeared to be a critical dose since it was non-cytotoxic according to the short-term MTS assay, but it markedly decreased the colony forming ability (Figure 2). Gene expression analysis was performed on cells treated both with SM30 and AS30 NPs and incubated for 3 h after treatment (short recovery time) and on cells treated with only AS30 NPs and incubated for 22 h after treatment (long recovery time), since their survival fraction was very similar to that of SM30 treated cells (Figure 2B).

**Figure 3:**
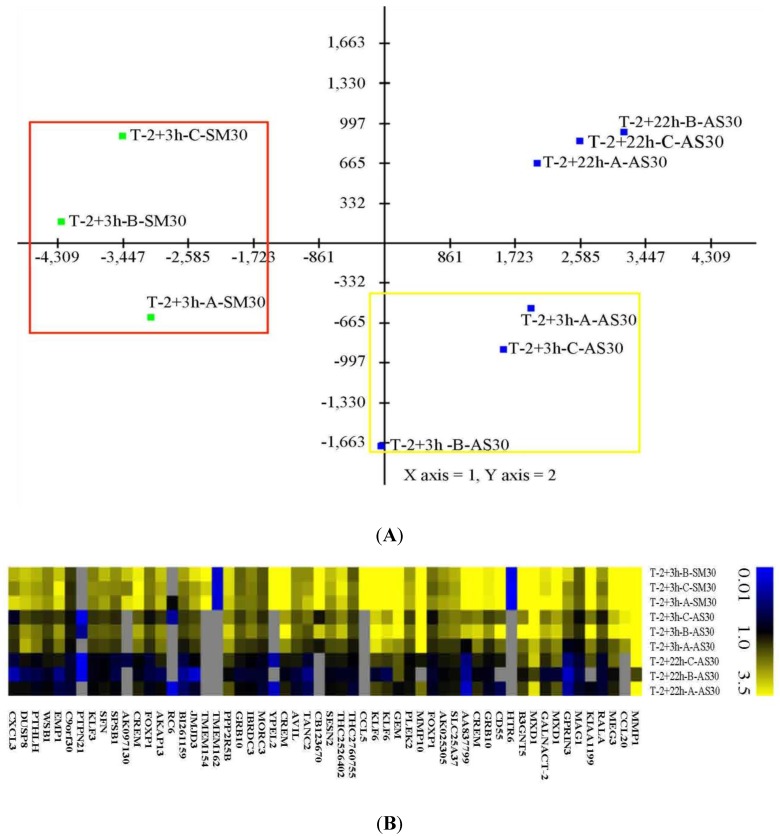
Microarray data analysis in A549 cells treated with SM30 and AS30 NPs. (**A**) Cell treatments and recovery times appear different from each other (green: SM30 NPs and blue: AS30 NPs). The red rectangle identifies the group of cells treated with smaller NPs (SM30) while the yellow one identifies the group of cells treated for the same time with bigger NPs (AS30). (**B**) Heat map of differentially expressed genes showing a decreased expression from SM30 treated cells to AS30 treated cells. Most of genes are involved in inflammatory response processes. T = Treated; 2 + 3 h and 2 + 22 h (2 h of treatment in serum-free medium containing 0.02 mg/mL NPs followed by 3 or 22 h of recovery time in complete medium). A, B, C indicates biological replicates.

The Principal Components Analysis (PCA) results indicated that cell treatments and recovery times are different from each other with the SM30 treatment evidencing a most distinct expression profile than AS30 treatments (Figure 3A). The first three components of PCA account for 98.6% of the observed_variance. The first two components, that account for the 87% of the observed variance, separate cells into two groups corresponding to treatment with smaller (SM30) and bigger (AS30) NPs showing their different transcriptional response (Figure 3A). The difference in size of SM30 and AS30, that accounts for a ~4-fold difference in surface area, allows a better discrimination between NP effects on transcriptional profile. In bacteria it has been observed that smaller nanoparticles have an easier time getting through the cell membrane than larger ones [46], therefore this could be an explanation for the differences in transcriptional responses we evidenced in human cells. Moreover, recovery time allows a different transcriptional behavior. Samples with a longer recovery time are completely separated from those with the shorter one (Figure 3A).

PCA analysis suggests the ability to identify gene marker useful in the discrimination of specific cell treatment and, therefore to monitor NPs effects. Statistically significant up-regulated genes in the 2 + 3 h treatment with smaller NPs (SM30) in comparison with AS30 NPs (Supplemental Table S1) are mainly involved in the program of the cell death and apoptosis, regulation of transcription and in inflammatory response (Table 3 and Supplemental Table S2). Genes up-regulated in response to bigger Ludox^®^ NPs (AS30) did not show such impact on cell inflammatory response but mainly on vesicle transport (Supplemental Table S2). In fact, altered transcripts enriched for the inflammatory response show a peculiar decreasing expression from cells treated with smaller NPs to the bigger ones (Figure 3B).

Considering that gene transcription was more altered following SM30 treatment we focused on such conditions to identify master regulator genes. We analyzed the interaction network composed by genes up-regulated after SM30 treatment (Supplemental Table S1). The resulting network appears complex (643 nodes and 2193 edges) (Supplemental Table S3 and Supplemental Figure S1) and hard to interpret. To gain insight into the network structure various topological parameters were calculated (Table 4); in fact, network statistics can be used as descriptive statistics for networks [42].

**Table 3:** GO terms of biological process significantly affected by SM30 NPs in A549 cells. Count = number of differentially expressed genes identified within each category.

GO.ID	Term	Count	*p* Value
GO:0042981	Regulation of apoptosis	29	2.9 × 10^−6^
GO:0043067	Regulation of cell death	29	3.5 × 10^−6^
GO:0006357	Regulation of transcription from RNA polymerase II promoter	25	3.8 × 10^−5^
GO:0006954	Inflammatory response	12	0.00436
GO:0009611	Response to wounding	16	0.00507
GO:0006952	Defense response	16	0.01816

**Table 4:** Summary of the principal topological parameters estimated for the network sustained by up-regulated genes in cells treated with SM30 NPs.

Topological Parameters	SM30 Network
Average clustering coefficient	0.651
Connected components	32
Avg. number of neighbors	6.278
Network radius	1
Network diameter	11
Network centralization	0.065
Network density	0.009
Network heterogeneity	0.997

According to centralization parameter the network appears decentralized (value for centralized network have to be close to 1). The network heterogeneity distribution has been the focus of considerable research in recent years revealing that biological networks tend to be very heterogeneous with the majority of nodes that tend to have very few connections [42]. This is in accordance with our results (network heterogeneity close to 1), with the number of connected components, diameter and the small number of neighborhood genes. Connected components indicates the connectivity of a network; a lower number of connected components suggests a stronger connectivity [47], while the network produced with the Agilent meta-search tool not presented a small number of connected components. It is important to identify highly connected “hub” genes/nodes because they play an important role in organizing the behavior of biological networks [48]. The degree of a node is the number of edges connected to the node and allows identifying hubs. In general, hub genes are master regulators and play important roles in the biology of the cell [49,50]. In the network, we define as hub nodes presenting a degree higher the nodes average degree (average degree = 19). 4.82% of the nodes have a degree higher than average (31 nodes with degree > 19 *vs.* 643 nodes; Supplemental Figure S1).

**Figure 4:**
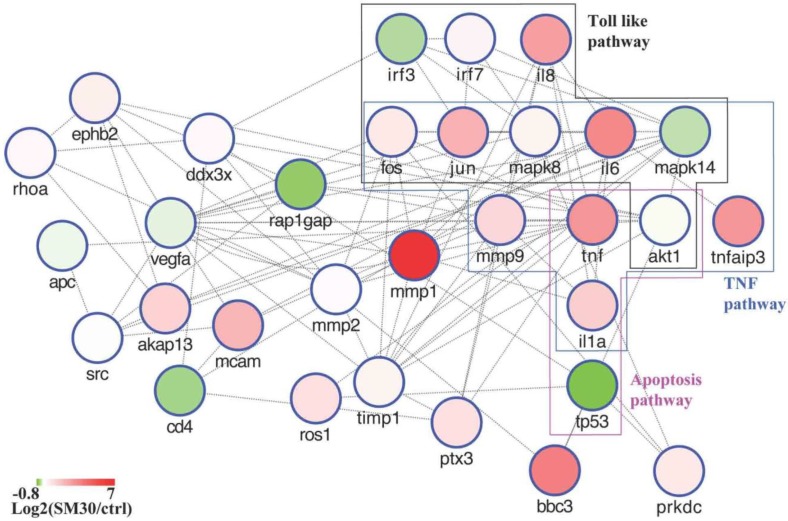
Regulatory network reconstructed using literature information for nodes with degree higher than 20. Nodes of the network are colored according to their expression in cells treated with SM30 while node border is colored according to node degree (blue is for degree higher than 20).

To resolve the function of “hub” genes we constructed a subnetwork containing hub interactions (Figure 4). 45% of most interconnected nodes (degree higher then 20) (Figure 4) were identified as up-regulated in cells treated with SM30. This result could sustain their importance in the processes activated by SM30 treatment. As evidenced in the Figure 4 a considerable number of hub genes are involved in inflammatory processes through the production of citokines. *Matrix metallopeptidase 1* (*MMP1*) is the gene most up-regulated in cells treated with SM30 NPs (133-fold against controls). Such strong gene activation was not seen in other conditions (Supplemental Table S1). *MMP1* is a hub component of the network describing gene interaction between most up-regulated genes following the A549 cells treatment with SM30 NPs (Figure 4). Matrix metalloproteinases play critical roles in inflammation, tissue remodelling and carcinogenesis of pulmonary tissue contacted by air pollutants [51]. The induction of the expression of *MMP1* by SM30 NPs and its hub position in the network can sustain the hypothesis that it could be involved in the health effects of NPs. Also diesel exhaust particles induce *MMP1* expression in A549 cells [52]. Another up-regulated gene in cells treated with SM30 NPs is *matrix metallopeptidase 10* (*MMP10*) (23.9-fold against controls) that was also altered by TiO_2_ 7 nm NPs [20]. The degree of *MMP10* in the network we constructed was 10 (Supplemental Figure S1). As mentioned before the identification of differentially expressed genes is a challenging approach influenced by the statistical tests adopted and on the threshold used to declare a gene significant [28]. To overcome this drawback and better understand functional effects driven by NPs we chose to perform a genome wide analysis based on gene sets. Using *a priori* biological information allows the analysis of pathways taking advantage of the ability to monitor the expression of all genes in the pathway and evidencing concurrent modifications. This approach avoids the simple expression match of small number of differential expressed genes in the pathways lacking the information about homogeneous gene expression variations.

### 3.4. Supervised Approach: Pathway Analysis

The pathway analysis approach evaluates gene expression profiles among related genes, looking for coordinated changes in their expression levels. Several implementations of pathway analysis are now available [29]. Here, we used the Reactome and KEGG databases [53,54] to identify gene pathways that are altered in cells treated with SM30 NPs (Table 5).

**Table 5:** Summary of GSEA analysis based on the Reactome database. Set size refers to the dimension of the pathway, and NTK (Normalized *T*-test of the kth gene set) is the observed value of the statistic as defined in the Graphite web tool. Negative NTK values indicate pathways inhibited in treated cells while positive values indicate pathways activated in treated cells.

Pathway	Set Size	NTk Stat	NTk *q*-Value
Activation of ATR in response to replication stress	33	−5.89	0
G2/M Checkpoints	37	−5.31	0
CDC6 association with the ORC: origin complex	10	−4.94	0
Activation of the pre-replicative complex	28	−4.87	0
E2F mediated regulation of DNA replication	30	−4.76	0
M Phase	96	−3.67	0
Association of licensing factors with the pre-replicative complex	14	−3.09	0.012315271
G1/S-Specific Transcription	17	−2.65	0.031397174
Synthesis of glycosylphosphatidylinositol (GPI)	15	−2.51	0.036945813
DCC mediated attractive signaling	11	2.37	0.048701299
Regulation of Complement cascade	10	2.41	0.044994376
Activation of Matrix Metalloproteinases	21	2.41	0.044994376
Acyl chain remodelling of PE	13	2.58	0.035714286
Activation of BH3-only proteins	16	2.58	0.035714286
Signaling by Robo receptor	24	2.65	0.031397174
Nucleotide-binding domain, leucine rich repeat containing receptor (NLR) signaling pathways	44	2.65	0.031397174
p38MAPK events	12	2.65	0.031397174
Acyl chain remodelling of PC	14	2.75	0.027472527
Chemokine receptors bind chemokines	27	2.88	0.020120724
GAB1 signalosome	71	2.88	0.020120724
Antigen Activates B Cell Receptor Leading to Generation of Second Messengers	18	3.09	0.012315271
Translocation of GLUT4 to the Plasma Membrane	47	3.09	0.012315271
Signalling to RAS	28	3.09	0.012315271
Cell junction organization	66	3.09	0.012315271
O-linked glycosylation of mucins	44	3.2	0
Interleukin-2 signaling	38	3.53	0
Signalling to ERKs	34	3.59	0
Downstream signal transduction	120	3.9	0
Glycerophospholipid biosynthesis	68	4.21	0
Cell-Cell communication	101	4.23	0
Signaling by ERBB4	106	4.45	0
TRAF6 Mediated Induction of proinflammatory cytokines	64	4.47	0
MyD88 cascade initiated on plasma membrane	73	4.56	0
Toll Like Receptor 10 (TLR10) Cascade	73	4.56	0
Toll Like Receptor 5 (TLR5) Cascade	73	4.56	0
TRAF6 mediated induction of NFkB and MAP kinases upon TLR7/8 or 9 activation	73	4.62	0
NFkB and MAP kinases activation mediated by TLR4 signaling repertoire	71	4.71	0
MyD88-independent cascade	76	4.71	0
Toll Like Receptor 3 (TLR3) Cascade	76	4.71	0
MyD88 dependent cascade initiated on endosome	74	4.72	0
Toll Like Receptor 7/8 (TLR7/8) Cascade	74	4.72	0
Toll Like Receptor 4 (TLR4) Cascade	92	4.76	0
Toll Receptor Cascades	105	4.79	0
Signaling by SCF-KIT	106	4.8	0
Activated TLR4 signalling	88	5.09	0
MyD88:Mal cascade initiated on plasma membrane	78	5.1	0
Toll Like Receptor 2 (TLR2) Cascade	78	5.1	0
Toll Like Receptor TLR1:TLR2 Cascade	78	5.1	0
Toll Like Receptor TLR6:TLR2 Cascade	78	5.1	0
Signaling by Interleukins	91	5.1	0

The most significantly inhibited pathways are the “Activation of ATR in response to replication stress”, “G2/M checkpoints”, together with other pathways involved in DNA replication and cell cycle.

**Figure 5:**
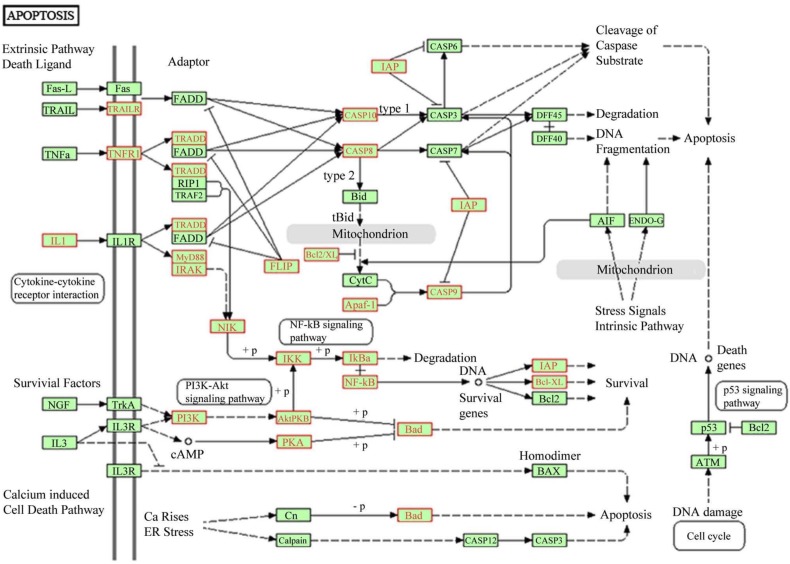
Scheme of apoptosis pathway according to KEGG database. In red are indicated genes altered after the treatment of A549 cells with SM30 NPs. According to Graphite web tool 32.5% of genes of the pathway were up-regulated after cell treatment (Supplemental Table S5).

The kinase ATR is an essential regulator of genome integrity [55] and cells having a defective G2-M checkpoint enter in mitosis with unrepaired DNA, leading to death after cell division [56]. The inhibition of these pathways suggests that cells exposed to NPs activate a program of cell growth arrest and apoptosis induction [57,58] in accordance with the inhibition of cell proliferation induced by silica NPs [59]. Pathway enrichment analysis using the KEGG database evidenced that most of the genes describing apoptotic process were altered (Figure 5 and Supplemental Table S4).

**Figure 6:**
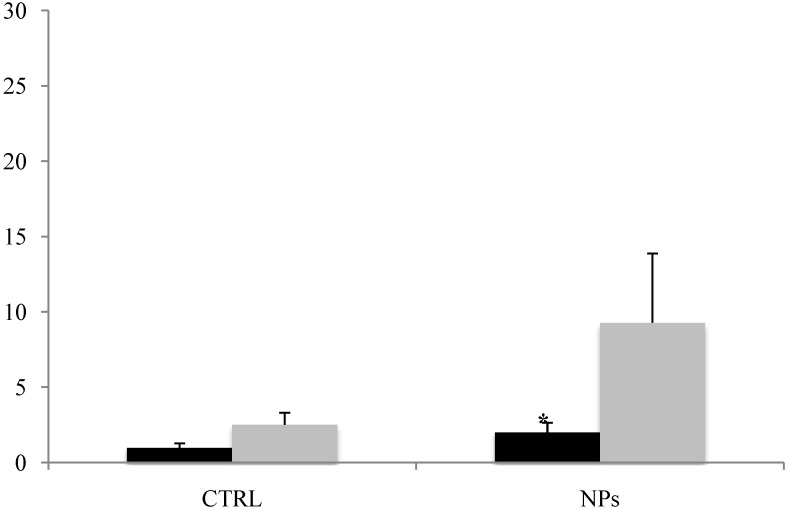
Apoptosis induction in cells treated with Ludox^®^ SM30 (0.04 mg/mL) for 2 h in serum-free medium, followed by a recovery of 3 h. After the recovery, the cells were double-stained with Annexin V-FITC/propidium iodide and analyzed by flow cytometry to detect cells in the early (black bars) or in the late stage (grey bars) of apoptosis.

Note: Data represent means ± SD (*n* = 3). (* *p* < 0.05, *t*-test; treated *vs.* control cells).

These finding are in accordance with the activation of the apoptotic program in A549 cells treated with SM30 NPs assessed through the Annexin V–FITC/propidium iodide double staining followed by flow cytometry analysis [33]. The assay shows the early activation of apoptotic processes (Annexin V staining) with the fraction of apoptotic cells that, after 3 h from treatment, reached 9%–11% (Figure 6).

**Figure 7:**
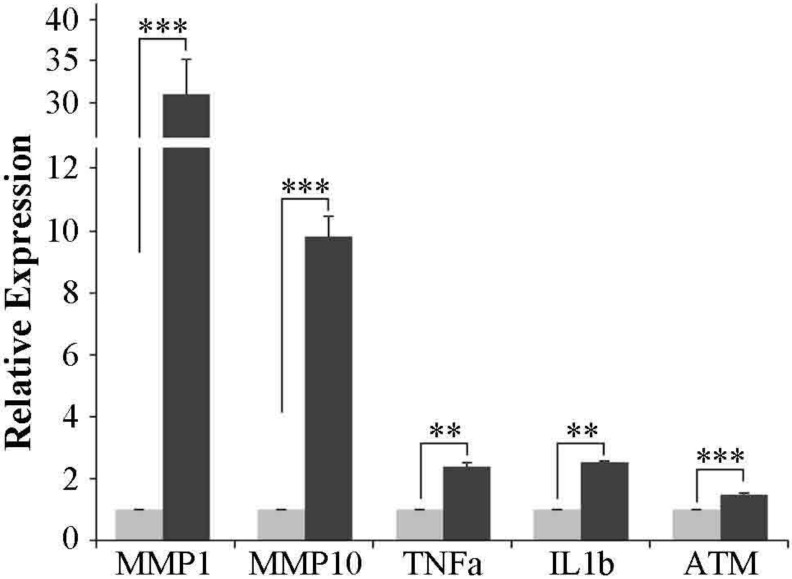
Microarray data validation by qRT-PCR in A549 cells treated with SM30 NPs. Values (fold change) are means ± S.D. of independent experiments performed in triplicate. The value “1” of control cells (light grey bars) is arbitrarily given when no change is observed.

Notes: *** *p* < 0.001, ** *p* < 0.01, *t*-test.

To confirm the activation of apoptotic pathway the expression of *TNF*, *IL1b* and *ATM* genes was tested by qRT-PCR (Figure 7). *ATM* is a central controller of genomic stability that phosphorylates downstream targets involved in cell cycle arrest, DNA repair and apoptosis. From microarray data *ATM* gene resulted slightly induced (1.3-fold) after exposure of A549 cells to SM30 NPs, and qRT-PCR confirmed a significant 1.5-fold induction *vs.* control cells (Figure 7). *TNF* and *IL1b* genes, two initiators of the apoptotic process, showed a significant up-regulation in SM30 treated cells (Figure 7). The microarray data from gene expression profiling were validated by qRT-PCR experiments also for *MMP1* and *MMP10* genes, whose expression level was significantly induced following NP treatment (Figure 7).

Through GSEA pathway analysis we evidenced that many immune-related pathways were significantly altered following NP exposure, such as those of toll like receptor signaling and interleukin signaling. In addition, the activation of the matrix metalloproteinases pathway evidenced the importance of these proteins in the cell response to Ludox^®^ NPs [51]. Gene-by-gene approach allowed the identification of *MMP1* and *MMP10* as marker genes of NP exposure (Figure 4 and Figure 7). In addition to *MMP1* and *MMP10*, other matrix metalloproteinases are activated by SM30 treatment, such as *MMP9* and *MMP2* (Figure 4). *MMP9* is induced by different metal and non-metal NPs [60,61,62] via oxidative signaling [63] through *fra-1*, a heterodimeric partner of *AP-1*, which binds to and activates the *MMP-9* promoter [64] or via toll like receptors [65]. This activation way appears to be the preferential one for lung cells treated with silica NPs; in fact, most of activated pathway in treated cells are involved in the toll-like receptor/MyD88 cascade (Table 5). Increasing *in vitro* approaches evidence the involvement of *MMP-9* in the apoptosis phenomena [66] through precise degradation of ECM proteins (e.g., laminin, fibronectin, vitronectin).

Gene set enrichment analysis does not take in account pathway topological information, which is essential to infer a more robust pathway activity. If transcript abundance ratios are altered, we expect a significant alteration not only of their mean expression levels, but also of the strength of their connections, resulting in pathways with completely corrupted functionality. Through CLIPPER analysis [44] we evidenced that apoptosis is a fundamental process activated by SM30 NPs (Supplemental Table S6). The activation of BH3-only proteins of the Bcl-2 protein family are essential for programmed cell death and are required for apoptosis induced by cytotoxic stimuli. These proteins have evolved to recognize distinct forms of cell stress. In response, they unleash the apoptotic cascade by inactivating the protective function of the pro-survival members of the Bcl-2 family and by activating the Bax/Bak-like pro-apoptotic family members [67].

## 4. Conclusions

In this work we applied a gene-by-gene and gene set analysis approaches to identify marker genes involved in the toxicity induced by Ludox^®^ silica NPs in human alveolar epithelial A549 cells. In both cases, we evidenced that 9-nm (SM30) *vs.* 18-nm (AS30) NPs give rise to a distinct gene expression profile and, the smaller the particles, the higher the effects on inflammatory and apoptotic cell responses. *MMP1* is the most up-regulated gene in cells treated with smaller NPs (SM30) and represents a master regulator of the constructed network. In addition, other matrix metalloproteinases genes such as *MMP10* and *MMP9*, and genes involved in the activation of apoptotic program (*TNFa*, *IL1b* and *ATM*) were up-regulated in response to SM30 treatment. Our results demonstrate the feasibility and usefulness of combining gene-by-gene and pathway analysis approaches in identifying new candidate genes whose expression is associated with specific experimental conditions. In particular, our results evidenced distinct transcriptional alterations in relation to different sized NPs at a dose of exposure which, according to our results of MTS assay, is non-cytotoxic and not able to discriminate between the two types of NPs. The data currently available are not enough to draw any conclusion about how the doses used in the present study may compare to possible real life exposures, for which data on concentrations and the form of NPs that are released into the environment are lacking. The NP concentrations indicated in the guidelines for occupational exposure [68] are lower than the dose used in the present study to assess gene expression changes. However, our data can be informative as well for the bioaccumulation occurring after exposure to Ludox^®^ silica nanoparticles.

## Supplementary Files

Supplementary File 1
