# Supplementary material for: Altered Gene Transcription in Human Cells Treated with Ludox® Silica Nanoparticles
Source: Int J Environ Res Public Health. 2014 Aug 28;11(9):8867–90. doi: 10.3390/ijerph110908867 (PMC4198995; doi:10.3390/ijerph110908867)
Supplement: Supplementary File 1 [file ijerph-11-08867-s001.zip › ijerph-56317-Supplemental Figures-xml.pdf]

Altered Gene Transcription in Human Cells Treated with Ludox® Silica Nanoparticles

---

Figure S1. Regulatory network reconstructed using literature information. Edges of the network are colored according to their expression in cells treated with SM30 (grey indicates that that gene is not differentially expressed) while node border color represents its degree (blue is for degree higher than 16). Nodes are cluster according to their degree indicated on rectangles on the right.

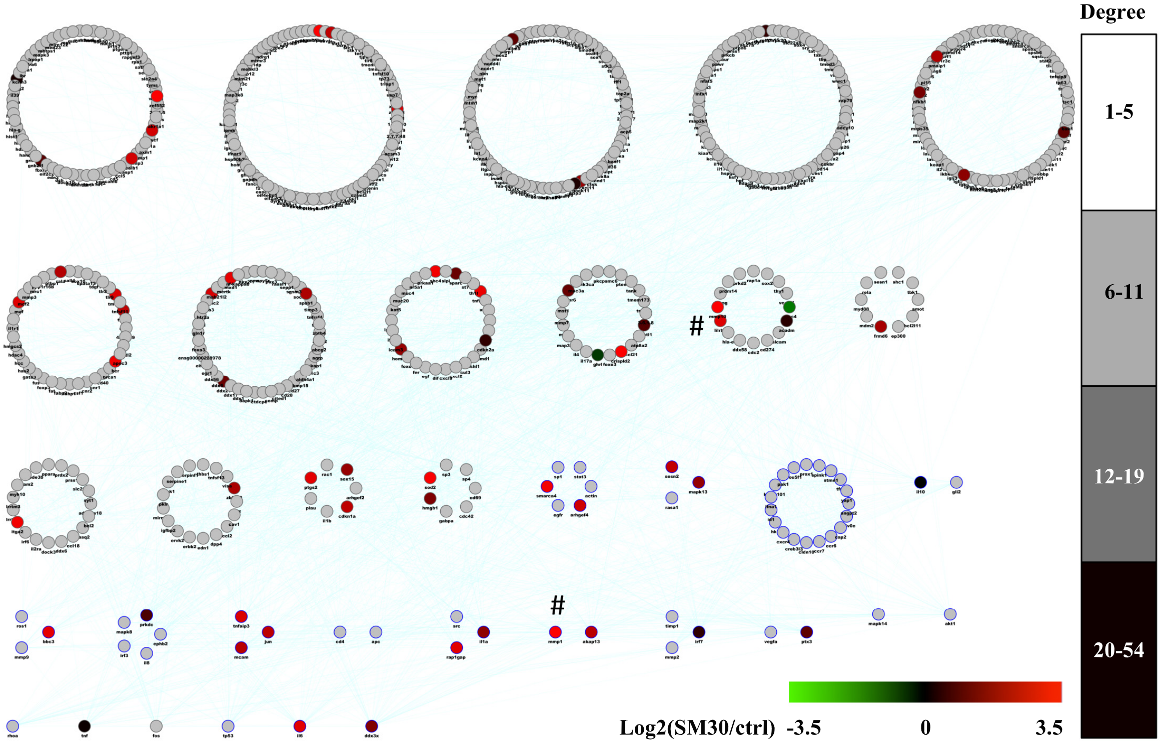

Note: # indicates *MMP1* and *MMP10*.

Figure S2. Cytotoxicity of Ludox® NPs AS30 and SM30 in A549 cells was assessed by MTS assay (**A**) and clonogenic assay (**B**), in relation to NP concentration and recovery time after the treatment (3 or 22 h).

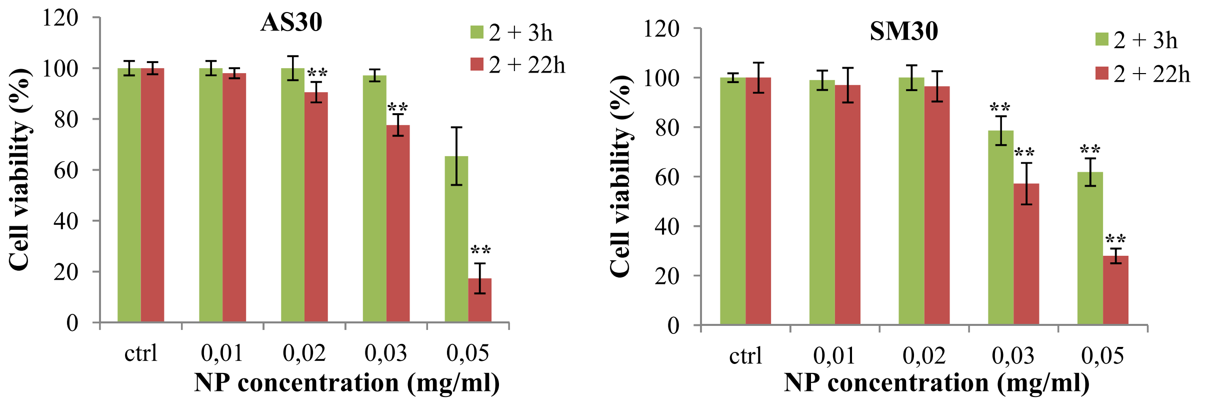

(**A**)

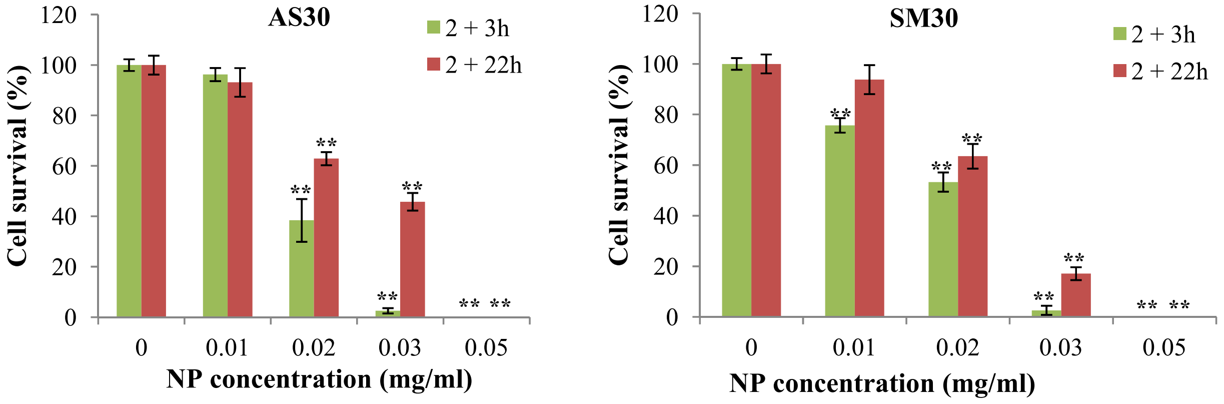

(**B**)

Notes: Data represent mean ± S.D. ($3 \leq n \geq 12$); ** $p \leq 0.01$ (NP-treated *vs.* controls).

© 2014 by the authors; licensee MDPI, Basel, Switzerland. This article is an open acess article distributed under the terms and conditions of the Creative Commons Attribution license (http://creativecommons.org/licenses/by/3.0/).
